# Electric-Field Response of Discotic Hexabenzocoronene (HBC) Liquid Crystals

**DOI:** 10.3390/molecules16119101

**Published:** 2011-10-31

**Authors:** Wenguang Wang, Xuying Liu, Jialing Pu

**Affiliations:** Laboratory of Printing and Packaging Material and Technology, Beijing Institute of Graphic Communication, Daxing District, Xinghuabeilu 25, Beijing 102600, China

**Keywords:** discotic liquid crystal, hexabenzocoronene, electric field response, phase transition

## Abstract

A HBC derivative bearing six branched, space-demanding, alkyl side chains containing ether linkages has been synthesized and its thermotropic properties were investigated by polarization optical microscopy (POM) and differential scanning calorimetry (DSC). To our surprise, this molecule can respond to electric fields, and the influences of alternating current (AC) and directing current (DC) electric field on the assembly of this molecule in liquid crystal cells were discussed.

## 1. Introduction

**Figure 1:**
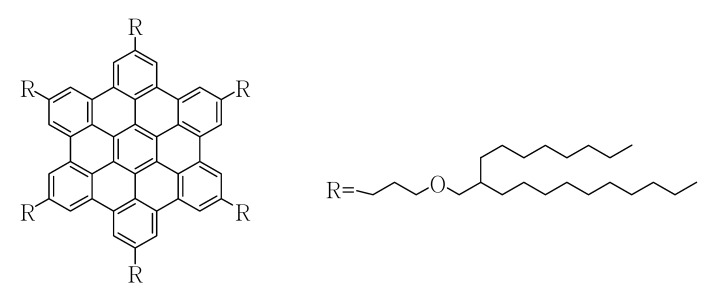
Structre of the synthesized HBC molecule.

**Scheme 1:**
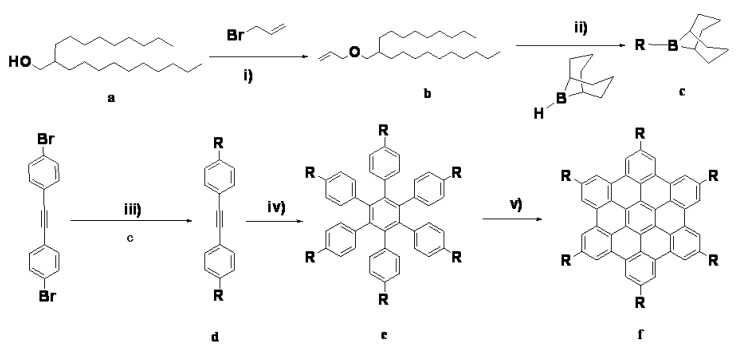
Synthesis of the target HBC molecule hexa(3-(2-decyldodecyloxy)propyl)HBC.

Discotic liquid crystals (LCs) capable of forming columnar mesophases [1,2,3,4] are of increasing interest attributable to their potential applications in molecular electronics or organic photovoltaics [5,6,7,8]. However, unlike rodlike LC molecules that align unidirectionally under the influence of an electric field to assemble controllably nematic and smectic phases as desired [9,10], discotic LC molecules often display stable alignments under due to their poor flowability under a 2D structural order of strong π-π stacking, so efforts to influence the alignment of discotic LCs using electric fields have floundered [5,6,11,12,13,14,15,16]. Some researchers have recently discussed the response of discotic LC assemblies to an applied electric field [14,15,16,17,18,19]. If semi-conductive discotic liquid crystals that assemble columnarly can be aligned likewise by an electric field, superior conducting electronic devices with suitably oriented pathways for high carrier transport would be developed [17]. Therefore, strategies to control the alignment of discotic LC molecules under an electric field are attractive. Here, we report the synthesis of a kind of discotic hexabenzocoronene derivative (Figure 1) which has six branched, space-demanding, alkyl side chains containing ether linkages following a slightly modified literature procedure (Scheme 1) [20].

## 2. Results and Discussion

**Figure 2:**
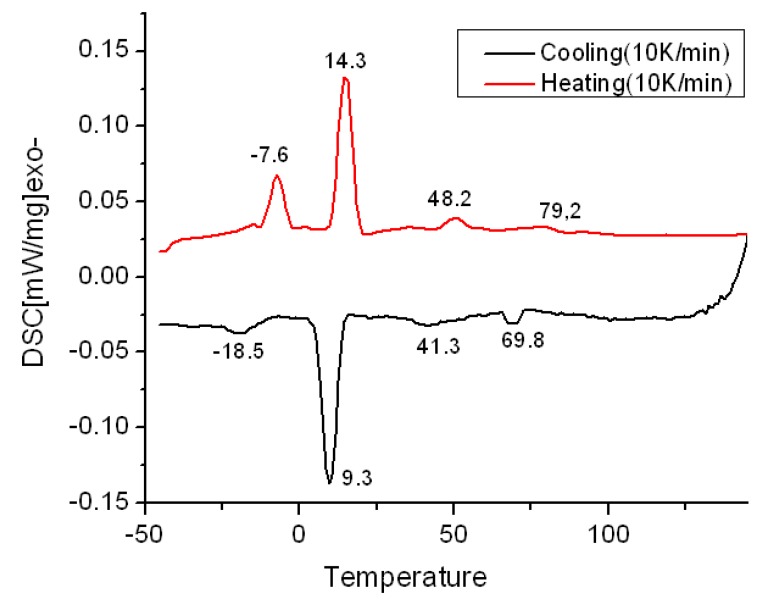
DSC traces during ﬁrst heating/cooling cycle of synthesized HBC.

**Figure 3:**
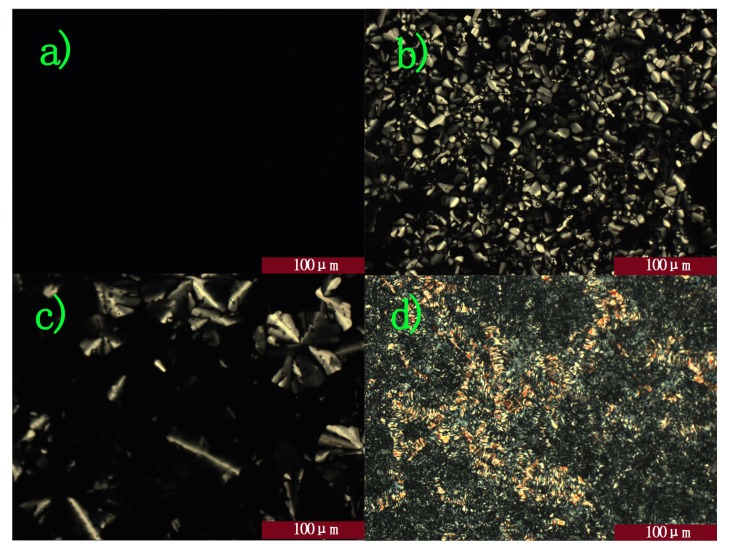
POM of HBC derivative. (**a**) at 85 °C with crossed polarizers; (**b**) 65 °Cwith crossed polarizers; (**c**) at 60 °Cwith crossed polarizers for two hours; (**d**) at 25 °Cwith crossed polarizers.

According to differential scanning calorimetry (DSC) analysis of this compound (Figure 2), this molecule self-assembles into LC mesophases over a wide temperature range, including room temperature. The observation of textures in optical microscope images reveals the mesophase behavior of the liquid-crystalline molecule (Figure 3). This compound entered an isotropic phase at 79.2 °C (Figure 3a) upon heating at the rate of 10 °C min^−1^. Upon cooling at the same rate, the typical fan-like texture was observed (Figure 3b) below the clearing point, which indicated a columnar phase. Then, when the temperature stayed at 60 °C about 2 h, skeleton textures began to appear (Figure 3c). Upon further cooling, an undefined texture appeared from 41.3 °C to room temperature (the lowest temperature accessible with our microscope).

**Figure 4:**
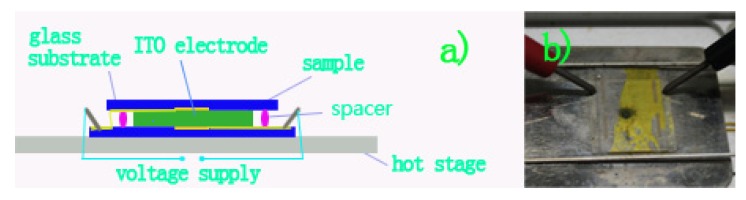
Photographs of the liquid crystal cell heated on hot stage with the application of vertical E-field.

For investigating the E-field responses, we prepared a sandwich-type cell with two parallel-oriented glass plates (15 μm spacer) partially coated with indium tin oxide (ITO) electrodes (1 cm^−2^) (Figure 4a). Liquid crystal HBC derivative was capillary-filled into the cell (Figure 4b).

**Figure 5:**
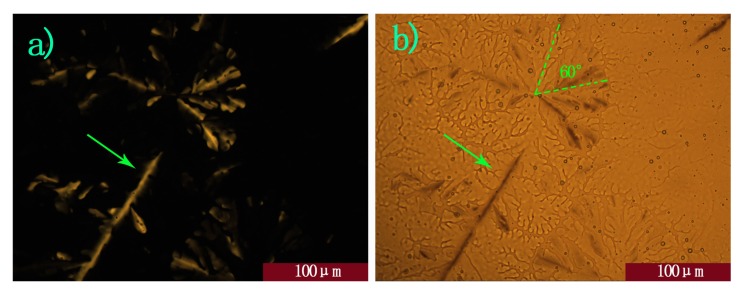
(**a**) and (**b**) optical microscope image with no E-field at 60 °C, (**a**) with (**b**) without cross-polarizers; The arrow indicates defects in the homeotropic order, and dashed lines indicates the propagation direction of the dendritic structures.

In detail, when no e-field was applied, the whole view was black above 79 °C, as shown in Figure 3a. As the temperature dropped, fan-like textures started to appear from 69 °C (Figure 3b) with cross-polarizers. Then, they grew increasingly larger and flocked together. More orderly Col_h_ mesophase domains formed when the temperature was stable for 2 h (Figure 5a). When cross-polarizers were removed, dendritic textures were observed (Figure 5b), which are characteristic for the homeotropic phase where the columnar axes are perpendicular to the substrate.

**Figure 6:**
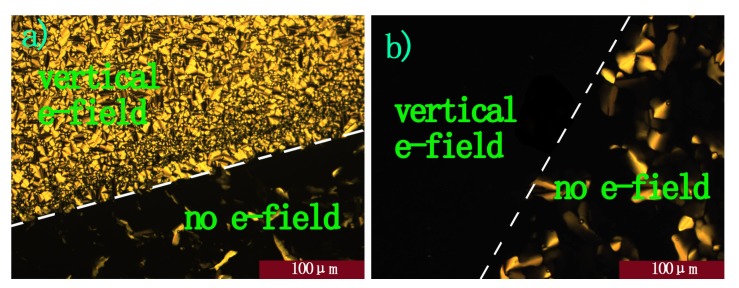
(**a**) and (**b**) POM image under cross-polarizers with vertical E-field; (**a**) DC; (**b**) AC, 50 Hz; sandwiched by glass plates with patterned ITO electrodes (15 μm spacer) under an applied electric field of 6 Vμm^−1^. The dashed lines represent the borders of the E-field operating and non-operating parts.

However, further research revealed that when an AC electric field (50 Hz, 6 Vμm^−1^) was applied to influence the alignment of the discotic liquid crystal, unprecedentedly, a great contrast appeared between operating and non-operating parts. The whole area of ITO was covered swiftly with fan-like textures (Figure 6a), which are typical of a disorder columnar mesophase, suggesting that the original order was disturbed by the time-varying electric field. In addition, when a lower electric field (50 Hz, 2 Vμm^−1^) was applied, it took relatively longer time to switch. Therefore, it provided direct evidence that if there exist incessantly varying electric fields around discotic columnar aggregates, the order degree of samples would decrease. We hypothesized that the inherent causes behind this phenomenon lie in the interactions between the time-varying electric field and the aggregates’ dipole moment and there may be a correlation between switch rate and the electric field strength. Another experimental result from charge carrier mobility tests showed that the mobilities decrease by orders of magnitude [21].

When DC voltage was applied to a sample film of this molecule during incubation in the cell at 60 °C, the film sample started to lose its characteristic birefringent texture in the E-field-exposed area sandwiched by the ITO electrodes (Figure 6b). In 2 h, the birefringency disappeared entirely in this area, clearly indicating that the LC columns were oriented homeotropically relative to the electrodes. In contrast, other areas without the E-field (*i.e.*, sandwiched by glass plates) remained birefringent. These observations demonstrated that the homeotropic orientation of the columns was driven by application of the DC electric field.

## 3. Experimental

### 3.1. General

Unless otherwise indicated, all starting materials were directly used without further purification. DSC was tested by P NETZSCH DSC200PC Calorimeter. ^1^H-NMR spectra were obtained on FT-80A spectrometer. POM images were measured by a Leica DM4500 polarization microscope.

### 3.2. Preparation of 4,4’-bis(3-(2-decyldodecyloxy)propyl)tolan ***(d)***

In a dry flask a 0.5 M solution of 9-BBN in THF (70 mL) was added slowly to compound **b** (prepared in our lab, 5.8 g, 20 mmol) under an argon atmosphere, and the mixture was stirred overnight. To this solution 3 M aqueous NaOH solution (13.0 mL) was added, followed after 15 min by 4,4′-bis(bromophenyl)acetylene (1.7 g, 5 mmol) and PdCl_2_(dppf) (0.2 g). The mixture was stirred under argon for 5 h and then extracted with CH_2_Cl_2_. After standard workup, the residue was purified by column chromatography to afford (2.8 g, 68%) of the tile compound as a colorless oil. ^1^H-NMR (300 MHz, CDCl_3_, 25 °C): δ 7.33 (d, ^3^*J* (H, H) = 8.2 Hz, 4H, CHarom), 7.07 (d, ^3^*J* (H, H) = 7.9 Hz, 4H, CHarom), 3.24 (t, ^3^*J* (H, H) = 6.0 Hz, 4H, O–CH_2_–), 3.12 (d, ^3^*J* (H, H) = 5.7 Hz, 4H, O–CH_2_–), 2.50 (t, ^3^*J* (H, H) = 7.4 Hz, 4H, α-CH_2_), 1.80–1.70 (m, 4H, β-CH_2_), 1.51–1.41 (m, 2H, CH), 1.32–1.10 (m, 68H, CH_2_), 0.80–0.70 (m, 12H, CH_3_).

### 2.2. Preparation of hexakis-4-(3-(2-decyldodecyloxy)propyl)hexaphenylbenzene ***(e)***

Co_2_(CO)_8_ (80 mg, 0.22 mmol) was added under argon to a degassed solution of compound **d** (2 g, 2.7 mmol) in dioxane (100 mL) in a 150 mL round bottomed flask equipped with a reflux condenser. After refluxing for 18 h, the solvent was evaporated under vacuum, and the residue was purified using column chromatography, yielding a colorless oil, yield: 60%. ^1^H-NMR (300 MHz, CDCl_3_, 25 °C): δ 6.76 (d, ^3^*J* (H,H) = 8.2 Hz, 12H, CHarom), 6.73 (d, ^3^*J* (H,H) = 8.2 Hz, 12H, CHarom), 3.32–2.18 (m, 24H, O–CH_2_–), 2.55–2.42 (m, 12H, α-CH_2_), 1.71–1.63 (m, 12H, β-CH_2_), 1.41 (br, 6H, CH), 1.25–1.10 (br, 204H, CH_2_), 0.79 (t, ^3^J (H,H) = 6.3 Hz, 36H, CH_3_).

### 2.3. Preparation of hexakis(3-(2-decyldodecyloxy)propyl)-hexa-peri-hexabenzocoronene ***(f)***

A flask was charged with compound **e** (0.10 g, 0.04 mmol) and dried CH_2_Cl_2_ (70 mL). A constant stream of argon was bubbled through the solution. Then, FeCl_3_ (0.9 g, 5.4 mmol) in CH_3_NO_2_ (5 mL) was added dropwise. After 10 min the product was extracted with CH_2_Cl_2_ and concentrated under reduced pressure. The residue was purified using column chromatography on silica gel with toluene as the eluent, and dried under vacuum to afford (0.08 g, 75%) of compound **f** as a yellow waxy solid. ^1^H-NMR (300 MHz, CDCl_3_, 25 °C): δ 8.83 (s, 12H, CHarom), 3.62 (t, ^3^*J* (H, H) = 6.1 Hz, 12H, OCH_2_), 3.36 (d, ^3^*J* (H,H) = 6.0 Hz, 12H, OCH_2_), 3.29–3.17 (br, 12H, α-CH_2_), 2.29–2.21 (m, 12H, β-CH_2_), 1.66–1.59 (br, 6H, CH), 1.41–1.07 (m, 204H, CH_2_), 0.79 (t, ^3^*J* (H, H) = 7.3 Hz, 36H, CH_3_).

## 4. Conclusions

We have investigated the influence of diverse electric fields on the alignment of a HBC derivative bearing six branched, space-demanding, alkyl side chains containing ether linkages. When an AC electric field was applied, the original order was disturbed by the time-varying electric field, indicating that alternating electric field is antagonistic to the order of its homeotropic alignment relative to the electrodes. More important, it was found that the switch rates are related to the electric field strength, which could be applied to the field of information recording. On the other hand, homeotropic orientation of the columns was strengthened to form a large-area unidirectional orientation by application of a DC electric field, a valuable discovery for the preparation of molecular electronics or organic photovoltaics [22,23,24]. Two-dimensional wide X-ray diffraction (2WXRD) investigations and charge carrier mobility determination are in progress for future research [21].
